# Formation of folates by microorganisms: towards the biotechnological production of this vitamin

**DOI:** 10.1007/s00253-018-9266-0

**Published:** 2018-08-02

**Authors:** José Luis Revuelta, Cristina Serrano-Amatriain, Rodrigo Ledesma-Amaro, Alberto Jiménez

**Affiliations:** 1 Metabolic Engineering Group, Department of Microbiology and Genetics, University of Salamanca, Campus Miguel de Unamuno, E-37007 Salamanca, Spain; 2 Imperial College Centre for Synthetic Biology and Department of Bioengineering, Imperial College London, London, UK

**Keywords:** Folate biofortification, Metabolic engineering, Microbial production, *Ashbya gossypii*, Vitamin B_9_

## Abstract

Folates (vitamin B_9_) are essential micronutrients which function as cofactors in one-carbon transfer reactions involved in the synthesis of nucleotides and amino acids. Folate deficiency is associated with important diseases such as cancer, anemia, cardiovascular diseases, or neural tube defects. Epidemiological data show that folate deficiency is still highly prevalent in many populations. Hence, food fortification with synthetic folic acid (i.e., folic acid supplementation) has become mandatory in many developed countries. However, folate biofortification of staple crops and dairy products as well as folate bioproduction using metabolically engineered microorganisms are promising alternatives to folic acid supplementation. Here, we review the current strategies aimed at overproducing folates in microorganisms, in view to implement an economic feasible process for the biotechnological production of the vitamin.

## Introduction

**Fig. 1:**
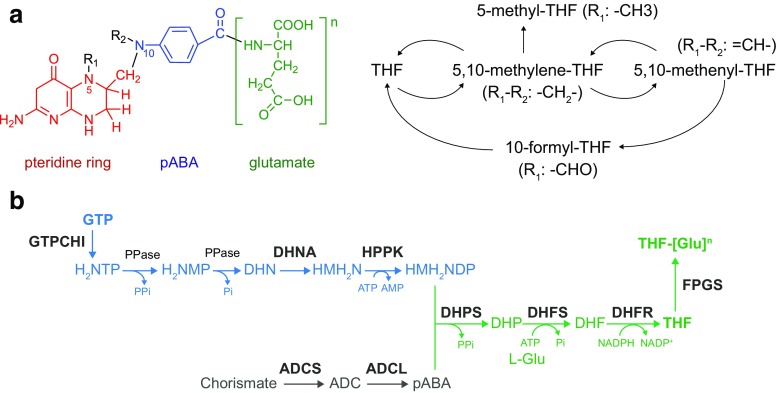
Folate structure and metabolic pathway. **a** Folates comprise a pteridine ring (red), a pABA molecule (blue) and a tail of gamma-linked L-glutamates (green). Different substituents at R_1_ and R_2_ characterize different vitamers which can be interconverted. **b** Schematic pathway of folate biosynthesis. Metabolites: H_2_NTP, into 7,8-dihydroneopterin triphosphate; H_2_NMP, 7,8-dihydroneopterin monophosphate; DHN, 7,8-dihydroneopterin; HMH_2_N, 6hydroxymethyl-7,8-dihydropterin; HMH_2_NDP, 6-hydroxymethyl-7,8-dihydroneopterin diphosphate; ADC, 4-amino-4-deoxychorismate; DHP, 7,8-dihydropteroate; DHF, 7,8-dihydrofolate; THF, tetrahydrofolate. Enzymes: GTPCHI, GTP cyclohydrolase I; PPase, phosphatase; DHNA, dihydroneopterin aldolase; HPPK, 2-amino-4-hydroxy-6-hydroxymethyldihydropterin pirophosphokinase; ADCS, aminodeoxychorismate synthase; ADCL, 4-amino-4-deoxychorismate lyase; DHPS, dihydropteroate synthase; DHFS, dihydrofolate synthase; DHFR, dihydrofolate reductase; FPGS, folylpolyglutamate synthase

Folates are a group of water-soluble compounds that are part of the B vitamin family (B_9_). They have a common chemical structure formed by a pteridine ring, a p-aminobenzoic acid, and one or more gamma-linked glutamates (Fig. 1). They function as coenzymes in C1 transfer reactions involved in the synthesis of purines, pyrimidines, and methionine, and the metabolism of amino acids (Tibbetts and Appling 2010).

This vitamin can only be synthesized de novo by fungi, some prokaryotes, and plants, and therefore, animals need to obtain it through the diet. Due to its involvement in such important metabolic pathways, its deficiency can cause several syndromes and diseases such as megaloblastic anemia, neural tube defects (NTDs), cardiovascular diseases, and cancer (Strobbe and Van Der Straeten 2017). Due to the difficulty of reaching the recommended daily intake (RDI) for this vitamin through the diet, in 1998 the US Food and Drug Administration (FDA) implemented a mandatory fortification program of enriched grain products to prevent NTDs (FDA 1996). Nowadays, the number of countries with a mandatory folic acid (FA) fortification program ascends to 58 (Arth et al. 2016), a measure which has proven to reduce in the incidence of NTDs.

In industry, FA is chemically synthesized, as there is no existing biotechnological process for its production at a large scale (Weimann et al. 2011). Although the synthetic form of the vitamin is not present in nature, it can be metabolized to bioactive forms by the action of the dihydrofolate reductase (DHFR). However, the human DHFR shows an extremely low rate of conversion of synthetic FA into bioactive vitamers and, therefore, administration of high concentrations of the synthetic form of this vitamin can lead to its accumulation in the bloodstream (Bailey and Ayling 2009). Consequently, this can mask a vitamin B_12_ deficiency because the symptoms are similar for both vitamins (Choi et al. 2014). This problem could be solved, however, by the fortification of food with the natural forms of folates. This, in addition to the rising interest in the use of more environmentally friendly processes in industry, has led to the development of metabolically engineered microorganisms and plants for the production of this vitamin.

While some reviews have focused on the biofortification of food crops with folates obtained through the metabolic engineering of plants (Strobbe and Van Der Straeten 2017), this mini-review concentrates on the metabolic engineering of microorganisms for their use in biotechnological processes in industry. First, the structure and the physiological role of this vitamin are described, as well as the biosynthetic pathway, focusing on the key enzymes susceptible to manipulation. Next, the bioavailability of synthetic and natural forms of folates from the diet is discussed, providing a general overview of the importance of the bioactive forms of folates. Also, biofortification is evaluated as a way to assure the correct daily intake of this vitamin. Then, the metabolic engineering of different bacteria and fungi for increasing the production of folates is reviewed, where recent work done for the development of industrial bioprocesses is emphasized. Finally, future perspectives in the use of synthetic biology for the development of metabolically engineered microorganisms for folate production are discussed. However, despite the works carried out so far, the industrial production of folates by microorganisms is still far from being economically feasible. Thus, more efforts are needed to increase the production levels of this vitamin.

## Folates: chemistry, biosynthesis, and physiological role

All folates have a common structure formed by a pteridine ring, a p-aminobenzoic acid (pABA) and a tail of gamma-linked L-glutamates, but can be differentiated. These forms differ in the oxidation state of the ring, where tetrahydrofolate is the most reduced form of folates; the C1 group that is bound to the positions N5 of the pteridine ring, and position N10 of the pABA moiety; and the number of glutamates that form the polyglutamate tail (Fig. 1).

Only plants and certain microorganisms possess the de novo pathway for the biosynthesis of folates, which has been very well-conserved throughout evolution. It comprises the synthesis of the pteridine ring from GTP, common precursor to the riboflavin biosynthetic pathway, its condensation with pABA, synthesized from chorismate, and the addition of a glutamate moiety (Rossi et al. 2016) (Fig. 1). The pteridine branch begins with the conversion of GTP into 7,8-dihydroneopterin triphosphate by the action of a GTP cyclohydrolase I (GTPCHI), followed by two dephosphorylation steps. The product, 7,8-dihydroneopterin, is converted into 2-amino-4-hydroxy-6-hydroxymethyl-7,8-dihydropterin by a dihydroneopterin aldolase (DHNA), which then is phosphorylated by a 2-amino-4-hydroxy-6-hydroxymethyldihydropterin pyrophosphokinase (HPPK). The pABA branch starts from chorismate, which is a product of the shikimate pathway. Two enzymes are involved in the first step, a glutamine amidotransferase, which generates ammonia from glutamine, and an aminodeoxychorismate synthase (ADCS), which transfers the ammonia group to chorismate forming 4-amino-4-deoxychorismate (ADC). The second step is catalyzed by a 4-amino-4-deoxychorismate lyase which converts ADC into pABA. A dihydropteroate synthase (DHPS) catalyzes the condensation of 2-amino-4-hydroxy-6-hydroxymethyldihydropterin diphosphate with pABA, forming 7,8-dihydropteroate. A glutamate is then added by a dihydrofolate synthase (DHFS) resulting in 7,8-dihydrofolate (DHF). DHF is reduced by a dihydrofolate reductase (DHFR), also present in animals, resulting in the first biological form of folate, tetrahydrofolate (THF) (Fig. 1). In bacteria, each enzyme is generally encoded by one gene, while in fungi and plants, it is common to find fused genes that result in multidomain enzymes. This is the case, for example, in *Saccharomyces cerevisiae* where the activities of DHNA, HPPK, and DHPS are contained in one enzyme encoded by *FOL1* (Lawrence et al. 2005).

Folates function as coenzymes in C1 transfer reactions and are involved in the synthesis of purines, pyrimidines, and methionine, the interconversion of serine and glycine, and glycine catabolism. The different forms of folates (i.e., THF, 5-methyl-THF, 5,10-methylene-THF, 5,10-methenyl-THF, and 10-formyl-THF) are interconverted by accepting or donating C1 groups during these reactions (Fig. 1). Folylpolyglutamate synthase (FPGS) catalyzes the addition of glutamate moieties to the different forms of folate, an event that is necessary for their retention in the cytosol or the mitochondria. Additionally, folate-dependent enzymes have more affinity for the polyglutamate folates, and therefore, this is the biologically active form of the vitamin (Shane 1989).

## Food availability and biofortification

Folates are mainly found in legumes (beans and peas) and green leafy vegetables. In addition, fruits, dairy products, poultry, and eggs are also important sources of folates (USDA National Nutrient Database for Standard Reference Legacy Release, April 2018). The recommended dietary allowance (RDA) for folates is expressed as dietary folate equivalents (DFE), which in turn is defined as 1 μg of food folate, to consider existing variations in bioavailability between different forms of folates. Hence, the US National Institutes of Health (NIH) recommends a folate RDA for adults of 400 μg DFE, while the folate RDA in the European Union (EFSA Panel 2014) is 240 μg DFE. A higher intake (600–1000 μg DFE) is advised for pregnant women (Rossi et al. 2016).

Synthetic FA and dietary folates differ considerably in terms of both bioavailability and bioaccessibility (see (Saini et al. 2016) and references therein). On one hand, only FA and monoglutamate folates can be absorbed in the gastrointestinal tract. Therefore, dietary folates, which mainly occur in the polyglutamate forms, must be processed by the folylpolyl-gamma-glutamyl carboxypeptidase (FGCP) into monoglutamates to be transported into the enterocytes. On the other hand, both FA and dietary monoglutamate folates must be transformed into 5-methyl-THF to be exported from the enterocytes to the blood vessels (Visentin et al. 2014). Hence, the main form of circulating folates in mammals is the 5-methyl-THF. Accordingly, several pre-absorptive and post-absorptive factors can significantly influence the bioavailability of ingested folates. Some examples of these factors include folate entrapment, both in plant cells and the food matrix, the gastric stability of folates, the fraction of polyglutamate forms and the genetic polymorphisms affecting folate metabolism (Gregory et al. 2005).

**Fig. 2:**
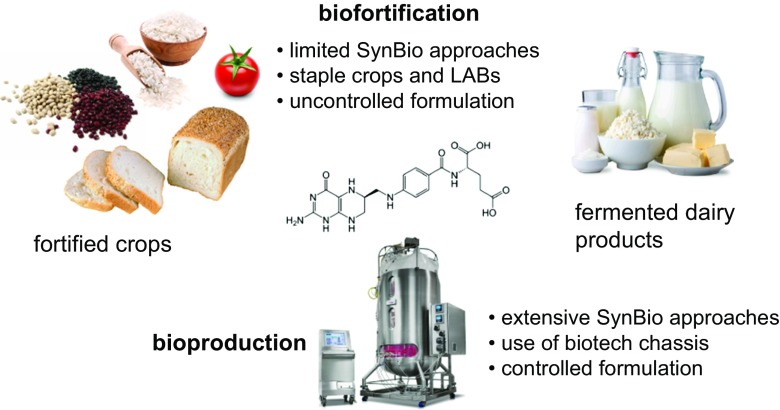
Folate food biofortification versus microbial folate bioproduction. Staple crops and LABs can be used for biofortification strategies. Microbial factories can be used for industrial bioproduction

As described above, clinical and epidemiological data show that folate deficiency is highly prevalent in many populations. For this reason, FA supplementation of foodstuffs has become mandatory in many developed countries (Arth et al. 2016). Moreover, there are strong economic reasons for the implementation of FA fortification programs in developing countries to reduce the mortality and morbidity associated with folate deficiency (Hoddinott 2018). Food fortification is generally achieved using synthetic FA, which is more stable than natural folates. However, natural folates are preferred to synthetic FA, as this may present possible unwanted health effects such as masking a vitamin B_12_ deficiency caused by the saturation of the DHFR activity or the increased risk of developing prostate and colorectal cancer (Saini et al. 2016). Hence, plant and microbial biofortification through classical breeding or genetic engineering can also be considered as an alternative to FA supplementation (Strobbe and Van Der Straeten 2017) (Fig. 2).

Folate biofortification in plants have been described in staple crops including rice, tomato, wheat, beans among others (Saini et al. 2016; Strobbe and Van Der Straeten 2017). Two general strategies have been carried out for plant biofortification by means of metabolic engineering. The first one involves the simultaneous overexpression of genes encoding GTP cyclohydrolase I (GTPCHI) and aminodeoxychorismate synthase (ADCS), which catalyze the first reactions of the pterin and pABA branches of the folate pathway, respectively (Fig. 1). The second strategy employs the overexpression of genes that contribute to increase folate stability (Fig. 1) (Strobbe and Van Der Straeten 2017). However, some of these approaches result in the enhanced levels of both intermediate pteridines and pABA (Díaz de la Garza et al. 2007; Saini et al. 2016). In addition, single overexpression of GTPCHI in tomato, the Mexican common bean, potato, and other types of plants also result in higher folate content, thus reflecting the complex regulation mechanisms of folate biosynthesis in different species (Strobbe and Van Der Straeten 2017).

Biofortification of dairy products and fermented foods using folate-producing LABs can be also considered as an additional way to achieve folate biofortification (Saubade et al. 2017). In this regard, the use of non-GMO bacterial strains requires the isolation of natural producers capable of synthesizing folates in fermented dairy products. Hence, some lactic acid bacteria (LAB) and bifidobacteria species are able to produce folates in fermented milk. However, the folate content is generally lower than 200 μg/L of fermented food, which is too low to satisfy the RDA (400 μg DFE) (Ayad 2009; Laino et al. 2014; Laino et al. 2012; Padalino et al. 2012). In addition, co-cultures of different LAB species have been used to increase the content of folates by about 30%, as compared to single cultures (Saubade et al. 2017). Nevertheless, serious concerns regarding folate stability during food processing must be considered in order to properly evaluate the use of biofortified dairy products and fermented foods, since the degradation and interconversion of folate vitamers can have a deep impact on the final concentration of folates (see Saubade et al. 2017, and references therein).

## Metabolic engineering for the overproduction of folates in microorganisms

Microbial metabolic engineering—the manipulation of metabolic pathways by genetic engineering—is a powerful tool for the bioproduction of high value molecules in microorganisms beyond their natural capacities. Such so-called engineered microbial cell factories have been successfully created for the overproduction of other vitamins such as B_2_, B_12,_ and C (Sauer et al. 2004; Rosa et al. 2013; Fang et al. 2017; Revuelta et al. 2017); but as earlier described, folates are still produced via chemical synthesis. However, several metabolic engineering approaches have been developed in both prokaryotic and eukaryotic microorganisms, which can serve as stepping stones towards a more sustainable production of vitamin B_9_ in the near future.

In the case of folate production, metabolic engineering can (1) enhance the metabolic fluxes towards folate production, increasing titers and yields, (2) control folate distribution maximizing the most desired (active/stable) form, and (3) maximize folate stability, which is known to be an important issue for folate storage.

Additionally, the approaches aimed at fortifying foods through the co-production of folates during food fermentation (work involving LABs or wine yeasts) can be differentiated from those intended to create a cell factory, in which folates are produced, extracted, and purified (Fig. 2); the latter approaches involve biotechnological chassis such as *Bacillus subtilis*, *Escherichia coli*, or *Ashbya gossypii*.

Most metabolic engineering efforts in prokaryotes have been carried out in the LAB *Lactococcus lactis* (Table 1), where the gene cluster involved in folate production was identified and some of the components subsequently overexpressed. The overexpression of the gene *folKE* (HPPK and GTPCHI activities) increases the extracellular folate production almost 10-fold and total folate almost 3-fold (Sybesma et al. 2003a). In addition, the overexpression of the endogenous *folKE* together with *folC*, encoding FPGS, increases the retention of folate in the cell. The overexpression of *folC* alone increases the polyglutamyl tail, thus generating the retention of all folates within the cell (Sybesma et al. 2003b). On the contrary, the overexpression of *folA*, encoding DHFR, decreases folate production, suggesting a feedback inhibition mechanism (Sybesma et al. 2003a). In another work, the same authors express a mammalian gamma-glutamyl hydrolase in *L. lactis* to convert polyglutamyl folate into monoglutamyl folate and to improve the excretion of bioavailable monoglutamyl folate into the fermentation broth (Sybesma 2003). In addition, the overexpression of the native GTPCHI in a specific riboflavin-producing strain enhances both vitamin B_2_ and vitamin B_9_ (Sybesma et al. 2004). The use of *L. lactis* in fermented food suggests these modified strains can be applied for biofortification, albeit regulatory restrictions exclude the use of GMO in foods. However, the levels achieved so far are still rather low (200 μg/L) (Sybesma et al. 2003b) and therefore more engineering approaches are still required.

**Table 1:** Folic acid yields of wild-type and engineered microorganisms

Microorganism	Titer mg/L	Application	Reference
*L. lactis*	0.2	Fortification	(Sybesma et al. 2004)
*Streptococcus thermophilus*	0–0.2	Fortification	(Padalino et al. 2012)
*B. subtilis*	0.03		(Zhu et al. 2005)
*B. subtilis BSZT0437*	0.16	Bioproduction	(Zhu et al. 2005)
*E. coli*	0.05		(Zhu et al. 2003)
*E. coli PB25*	0.27	Bioproduction	(Zhu et al. 2003)
*Bifidus adolescentis*	0.11	Fortification	(Pompei et al. 2007)
*S. cerevisiae Enoferm M2*	0.005	Fortification	(Walkey et al. 2015)
*S. cerevisiae*	0.36	Fortification	(Hjortmo et al. 2008)
*A. gossypii*	6.59	Bioproduction	(Serrano-Amatriain et al. 2016)

The biotechnological workhorse *B. subtillis* has also been engineered to increase folate production by combining theoretical flux analysis and metabolic engineering (Zhu et al. 2005). The best generated strain presented an inducible pyruvate kinase, overexpressing the *E. coli aroH* (2-dehydro-3-deoxyphosphoheptonate aldolase, involved in pABA synthesis), and increased the transcription and translation of genes within the folic acid operon. Such strain reached a production of 163 μg/L of folate (Table 1).

In addition, the model organism *E. coli* has also been engineered to overproduce folate by deletion of the pyruvate kinase (PYK) gene and redirecting the flux towards the synthesis of the basic metabolic precursors phosphoenolpyruvate and erythrose-4-phosphate (Zhu et al. 2003). This reached a production of 275 μg/L (Table 1).

In eukaryotic microorganisms, the folate production pathway has been characterized in the model organism *S. cerevisiae* (Berglez et al. 2005; Cherest et al. 2000; Nardese et al. 1996). In addition, this yeast has been manipulated to increase folate concentration during wine fermentation (Liu et al. 2016; Walkey et al. 2015). The overexpression of the endogenous gene *FOL2* has been found to be the most limiting step in folate overproduction (Liu et al. 2016; Walkey et al. 2015). Folate synthesis in *S. cerevisiae* has also been enhanced by optimizing media composition, reaching a production of 360 μg/L (Hjortmo et al. 2008) (Table 1).

**Fig. 3:**
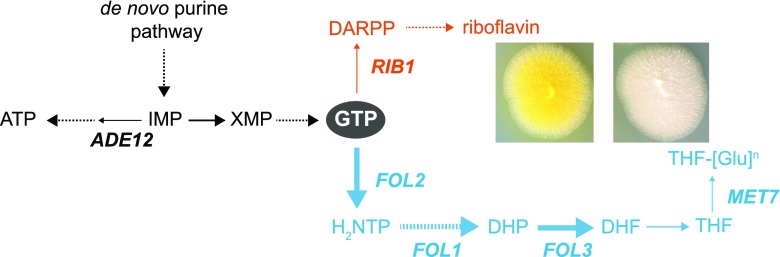
Metabolic engineering for folate bioproduction in *A. gossypii*. Increased availability of GTP for the biosynthesis of folate is achieved by reducing competing pathways: gene deletion of *ADE12* and gene underexpression of *RIB1*. Boosting folate production is accomplished by gene overexpression of *FOL* genes. Switching metabolic flux towards either riboflavin production or folate production results in super yellow strains or light yellow strains, respectively. Dashed lines indicate multistep pathways; thin lines indicate reduction of metabolic flux; thick lines indicate increase of metabolic flux

In a more recent approach, *A. gossypii* was engineered for folate production (Serrano-Amatriain et al. 2016). *A. gossypii* is a filamentous fungus that naturally overproduces riboflavin (vitamin B_2_) and, after mutagenesis and several rounds of rational engineering, is now one of the major industrial producers of this vitamin (Revuelta et al. 2017). Interestingly, the synthesis of riboflavin and folates present a common precursor (GTP), indicating that *A. gossypii* could be a good producer of folates (Fig. 3). *A. gossypii* can naturally synthesize 40 μg/L of folates and after metabolic engineering is able to reach 6595 μg/L (146 times more), which is the highest production titer ever reported (Table 1). This was achieved by firstly overexpressing the three endogenous *FOL* genes (*FOL1*, *FOL2*, *FOL3*), which increases the initial production 16-fold. Subsequently, the deletion of the *MET7* gene encoding FPGS increases folate production by more than 5.7-fold. As described above, FPGS catalyzes the polyglutamylation of folates in their gamma-carboxyl residue, and its inhibition is thought to decrease intracellular retention abolishing the feedback regulation. The elimination of competing pathways, such as riboflavin (by the downregulation of *RIB1*) and adenine (by gene deletion of *ADE12*) also enhances folate production. Finally, the combination of these modifications in one single strain (*ade12*∆, *met7*∆, *P*_*RIB7*_*-RIB1*, *P*_*GPD1*_*-FOL2-3*) generates the best folate producer reported to date (Serrano-Amatriain et al. 2016) (Table 1).

As previously described, plants are the most important natural sources of folates; therefore, metabolic engineering approaches have been carried out in crops with the aim of increasing folate content. A recent review by Stobbe and Van der Straeten (folate biofortification in food crops) summarizes most of the engineering approaches in plants (Strobbe and Van Der Straeten 2017). Interestingly, knowledge from these works can be applied to the microbial folate pathway. Some of the possible targets could involve the overexpression of folate binding proteins (FBPs), which are thought to increase folate levels by promoting their sequestration and reducing negative feedback regulations and to augment folate stability during storage. The expression of the folate binding glycine N-methyl-transferase (GNMT) from rat liver enhanced folate production in rice 8.8-fold (Abilgos Ramos 2010). Other potential targets are those enzymes involved in the interconversion between folate forms, which also could increase stability of the folate pool. For example, a mutated formyl-THF cycloligase (5-FCL), the sole enzyme known to consume 5-formyl-THF, prompts the accumulation of this stable form of folate (Goyer et al. 2005). In addition to rational strategies in plants, there have been many approaches to improve folate production through breeding programs. QTL analysis could reveal potential new targets for enhancing folate production in plants whose homologs in microorganisms, if any, could lead to improved microbial cell factories.

In recent years, metabolic engineering has grown as a field in parallel with the development of synthetic biology—a discipline that attempts to bring engineering concepts such as reproducibility, standardization, or modularity to biology. Examples of this involve the use of modular DNA assembly techniques that can facilitate the generation of engineering libraries of variants for the optimization of metabolic pathways or genome editing techniques such as CRISPR-Cas9, which can improve the efficiency of genetic engineering. These and other new techniques, together with the identification of novel target genes for engineering are expected to further boost the biotechnological production of folates. These techniques that allow the fine control of metabolic pathways can also be used to further study well-known overexpression targets in the folate pathway. For example, the co-expression of the pABA and pteridine branches of folate biosynthesis could be optimized by controlling the promoter strength of the enzymes of each pathway. This in turn would allow the most favorable balance for maximizing folate production to be identified while reducing undesired intermediates, limiting the transport of some of the accumulated intermediates between different compartments.

## Conclusions and future prospects

Folate deficiency continues to be a health problem in many overpopulated, war-ridden, poverty- or famine-stricken countries, as well as in some population sectors in developed, high-income countries. Two main strategies to increase the folate content of foods are being developed: biofortification and supplementation. The biofortification of food crops and fermented foods forms part of an integrated food-systems approach for reducing malnutrition, and efficiently targets the poorest people and rural households, improving family nutrition and health in a sustainable way. However, biofortification does not always assure healthy individuals in developed countries are receiving a sufficiently balanced natural daily folate supply, due to current food habits, the preference for processed foods, and preservation and cooking methods.

By contrast, supplementing foods with folates can be easily adapted to modern food technological processes which allows the final concentration of the vitamin in the commercial processed product to be efficiently controlled, as well as the precise definition of its formulation (i.e., vitamer composition, bioavailability, stability, etc.). Indeed, folate supplementation of foods, either voluntary or mandatory, has been implemented by most countries to efficiently fight folate deficiency (Arth et al. 2016). Unfortunately, all the vitamin B_9_ commercially available for food supplementation is chemically synthesized FA, and may present some drawbacks. Several studies seem to raise doubts regarding the safe use of chemically synthetized FA in foods, whereas natural folates do not cause such adverse health effects in individuals (Rossi et al. 2016; Saini et al. 2016).

For this reason, research initiatives are being carried out to evaluate the potential of natural folate production by microbial fermentation. In addition, microbial production is a sustainable technology based on renewable resources, and can be managed to produce an optimal mix of folate vitamers in an economically favorable way. Although a limited number of studies using metabolically engineered industrial microorganisms (*E. coli*, *B. subtilis*, LABs, and the fungus *A. gossypii*) has so far been reported, they do demonstrate the feasibility of the biotechnological approaches for industrial folate production (Hjortmo et al. 2008; Serrano-Amatriain et al. 2016; Sybesma et al. 2003a; Sybesma et al. 2003b; Zhu et al. 2003; Zhu et al. 2005).

Despite the remarkable improvements in folate production that have been achieved, the fermentation process is not competitive as yet with the chemical synthesis. Future research should thus focus on the following points: (i) understanding the complex regulatory mechanisms governing the enzymatic activities involved in the folate pathway; (ii) flux metabolic analysis to uncover possible bottlenecks and to channeling pABA and pteridine substrates towards the folate biosynthetic pathway; (iii) the blocking of chorismate-consuming pathways to enhance the synthesis of the limiting pABA substrate; (iv) the characterization and engineering of folate eukaryotic transporters to facilitate the import of the pABA and pterin substrates into the mitochondria, where the synthesis of folates takes place; and (v) the optimization of the fermentation conditions and further development of downstream processes for the recovery and purification of the product.
